# Data Evaluation of a Low-Cost Sensor Network for Atmospheric Particulate Matter Monitoring in 15 Municipalities in Serbia

**DOI:** 10.3390/s24134052

**Published:** 2024-06-21

**Authors:** Danka B. Stojanović, Duška Kleut, Miloš Davidović, Marija Živković, Uzahir Ramadani, Maja Jovanović, Ivan Lazović, Milena Jovašević-Stojanović

**Affiliations:** VIDIS Centre, Vinča Institute of Nuclear Sciences—National Institute of the Republic of Serbia, University of Belgrade, 11000 Belgrade, Serbia; duska@vin.bg.ac.rs (D.K.); davidovic@vin.bg.ac.rs (M.D.); marijaz@vin.bg.ac.rs (M.Ž.); uzahir@vin.bg.ac.rs (U.R.); majaj@vin.bg.ac.rs (M.J.); lazovic@vin.bg.ac.rs (I.L.); mjovst@vinca.rs (M.J.-S.)

**Keywords:** air pollution, low-cost sensors, particulate matter, sensor network, sensor network metrology

## Abstract

Conventional air quality monitoring networks typically tend to be sparse over areas of interest. Because of the high cost of establishing such monitoring systems, some areas are often completely left out of regulatory monitoring networks. Recently, a new paradigm in monitoring has emerged that utilizes low-cost air pollution sensors, thus making it possible to reduce the knowledge gap in air pollution levels for areas not covered by regulatory monitoring networks and increase the spatial resolution of monitoring in others. The benefits of such networks for the community are almost self-evident since information about the level of air pollution can be transmitted in real time and the data can be analysed immediately over the wider area. However, the accuracy and reliability of newly produced data must also be taken into account in order to be able to correctly interpret the results. In this study, we analyse particulate matter pollution data from a large network of low-cost particulate matter monitors that was deployed and placed in outdoor spaces in schools in central and western Serbia under the Schools for Better Air Quality UNICEF pilot initiative in the period from April 2022 to June 2023. The network consisted of 129 devices in 15 municipalities, with 11 of the municipalities having such extensive real-time measurements of particulate matter concentration for the first time. The analysis showed that the maximum concentrations of PM_2.5_ and PM_10_ were in the winter months (heating season), while during the summer months (non-heating season), the concentrations were several times lower. Also, in some municipalities, the maximum values and number of daily exceedances of PM_10_ (50 μg/m^3^) were much higher than in the others because of diversity and differences in the low-cost sensor sampling sites. The particulate matter mass daily concentrations obtained by low-cost sensors were analysed and also classified according to the European AQI (air quality index) applied to low-cost sensor data. This study confirmed that the large network of low-cost air pollution sensors can be useful in providing real-time information and warnings about higher pollution days and episodes, particularly in situations where there is a lack of local or national regulatory monitoring stations in the area.

## 1. Introduction

Air pollution, including both particulate matter (PM) and gas-phase pollutants, continues to be a worldwide hot topic since it is associated with various negative effects on human health [1]. Outdoor air pollution, consisting of gaseous pollutants and particulate matter, was designated by the International Agency for Cancer Risk (IARC) as a Group 1 carcinogenic substance, i.e., proven human carcinogen [2]. Ambient PM was also evaluated, independently of gas-phase pollutants, and classified as a Group 1 carcinogenic substance [2]. In most counties, National Air Quality Standards for main ambient pollutants are much higher than the WHO Air Quality Guidelines values [3].

Air pollution is a complex phenomenon, originating from multiple anthropogenic and natural sources. Anthropogenic sources include vehicular transport, domestic heating, energy generation, and industrial production. This highlights the need for improvements in monitoring air pollution because of its highly variable spatial and temporal patterns. National and local air quality monitoring networks monitor and measure air pollutants by methods with prescribed accuracy and precision, using high-end monitors and sophisticated quality assurance and quality control systems that are very expensive to establish, run, and maintain. This ensures comparability of measurements across different geographical areas and meteorological and climatic conditions. The number of air quality monitoring stations is limited because of the high price of reference- and equivalence-grade monitors and the high costs of qualified personnel that perform monitoring and maintain instruments. However, for personalized information, it is necessary to have access to air quality data with higher temporal and spatial resolution. Over the past decade, both the scientific community and citizens have recognized the great potential of the emerging paradigm of low-cost (LC) air quality sensors and documented the pros and cons of their use [4,5]. These sensors are small in size, modest in price, easy to handle, have fast response [6], and can be relatively easily deployed in a dense sensor network. This improves the spatial resolution of AQ measurements as they are widespread over cities [7,8,9], suburban [10] and rural areas, larger spaces [11,12], and hard-to-reach areas [12,13,14,15], which is their clear advantage over automatic monitoring stations. However, the performance of low-cost sensors needs to be carefully monitored as it can vary from sensor to sensor, which makes it necessary to examine the data quality of each node both during continuous use and before deployment [16]. Additionally, performance can vary spatially and over longer periods of time, as it depends on both short- and long-term exposure of the sensor to various environmental influences such as atmospheric composition and meteorological conditions [17]. Some of these performance issues can be alleviated by using a larger number of sensors connected in a network and applying methods and principles of sensor network metrology [18]. In this way, larger, richer, and more open data information can be obtained and used not only for mapping the pollutants but also for identifying sources, tracking changes, and predicting extreme air quality events [17]. 

The application of low-cost air quality monitoring networks has substantially grown over the last decade because of technological advances in the production of cheap and portable air pollution sensors [19,20,21,22,23,24]. Recent review papers [25,26] analysed practical aspects of outdoor air quality low-cost sensor networks, focusing on gas pollutant networks (analysed a total of 60 LCS networks targeting outdoor environments all over the world) and Carotenuto et al. [26] including both gas and PM sensor networks. In the open-access literature, Carotenuto et al. identified a total of 111 low-cost air quality monitoring networks for campaigns that had a duration from 3 months up to 4 years that were performed in the period 2014–2022. In the framework of AQ low-cost sensor networks, the most represented was atmospheric pollution of particulate matter fractions PM_10_ (13.3%), PM_2.5_ (41.2%), and PM_1_ (2.2%). The networks mainly consisted of 3–10 devices (52 LCS networks) and 10–100 devices (50 LCS networks), and only 7 had 100–500 nodes, with 2 networks having more than 2000 nodes [27]. Based on these results, it can be concluded that this puts a network built under the UNICEF initiative into a group of the 10% largest LCS network studies. 

Sensor networks, when deployed with a sufficient number of nodes, increase the often-limited spatial information on air quality conditions provided by conventional monitoring networks. However, the use of low-cost air quality sensors still has many limitations, mostly related to the reliability of their measurements and quality control of the large amount of data they provide [26]. Thus, to deploy a large-scale sensor network and meaningfully use the generated data, it is important to formulate standard operating procedures for assessing the short- and long-term performance of low-cost sensors and performance metrics that supplement the reported measurement results [28]. In addition, different approaches to evaluating data from low-cost sensors in air quality models have been proposed to increase spatial and temporal resolution [29,30,31]. It is also important to strike the right balance between network size and cost of calibration since if the network is too dense, the cost of calibration and maintenance can quickly become much larger than the initial cost of installation, thus defeating its original purpose. Therefore, the data have to be as accurate as they can, without the need for regular on-site calibration, because the necessary network scale may be very large, containing hundreds or more instruments [32,33]. Last, but not least important, the goal of such measurements is to engage communities in identifying concerns and solutions that can support adequate decision-making by institutions responsible for air quality control [34].

In this study, we present the results of a low-cost sensor network implementation that includes 43 locations in 15 municipalities in central and western Serbia in the period from 1 April 2022 to 30 June 2023. Before deployment in schools across the 15 municipalities, all sensors passed the procedure of collocation calibration at the automatic monitoring station. We also show daily PM_2.5_ and PM_10_ episodes and compare them with the Serbian Environmental Protection Agency (SEPA) open data for 4 out of the 15 municipalities in which automatic stations equipped with PM monitors exist. 

This paper is structured as follows. In Section 2, we describe the existing SEPA monitoring network and newly deployed low-cost sensor network. The results and a discussion are given in Section 3. Finally, the main conclusions of this study are given in Section 4.

## 2. Materials and Methods

### 2.1. Low-Cost Sensor Network

The sensor network, established under the “Schools for Better Air Quality” UNICEF Serbia pilot initiative, used a low-cost sensor node equipped with a PMS7003 air dust sensor for measurements of particulate matter fractions (PM_10_, PM_2.5_ and PM_1_), with a node specifically developed in the framework of the UNICEF initiative. As reported in a number of publications, the PMS7003 sensor has been successfully used for the detection of PM_10_, PM_2.5_, and PM_1_ in indoor and outdoor environments. PMS7003 belongs to the family of Plantower models (PMS1003, PMS3003, PMS5003, PMS7003, and PMS-A003) that have lower prices compared with other low-cost PM sensors with similar characteristics [35,36,37,38]. UNICEF nodes upload data into the cloud backend, calibration coefficients are applied at the server side, and near real-time data about air quality is made publicly available on a dedicated website.

**Figure 1:**
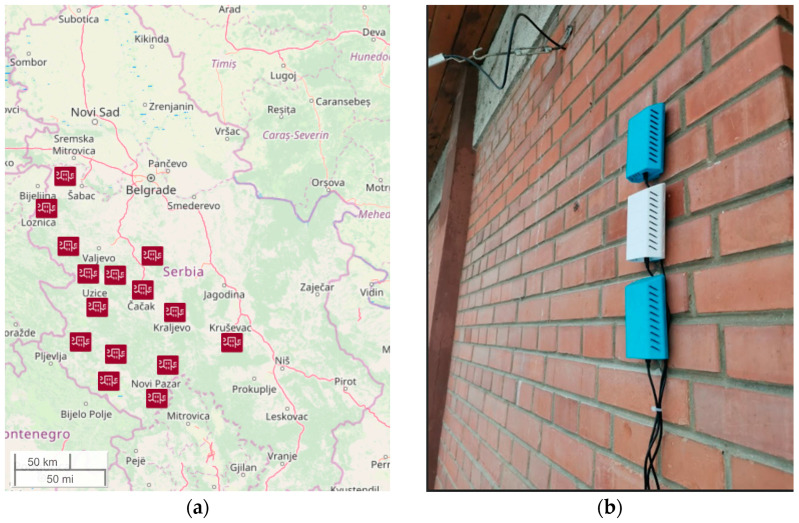
(**a**) Map of Serbia with the position of 15 municipalities that participated in this study. (**b**) An example of low-cost sensor positioning at one of the measuring sites. Note the redundant nodes, which increase the reliability of the network.

The low-cost sensor network consisted of 129 devices at 43 locations in 15 municipalities in central and western Serbia (the basins of two rivers including Velika Morava and Zapadna Morava), as shown in Figure 1a, including Užice (M1), Kraljevo (M2), Kruševac (M3), Čačak (M4), Gornji Milanovac (M5), Novi Pazar (M6), Raška (M7), Kosjerić (M8), Loznica (M9), Osečina (M10), Priboj (M11), Bajina Bašta (M12), Bogatić (M13), Nova Varoš (M14), and Prijepolje (M15). Network nodes were made in the framework of the UNICEF initiative, equipped with low-cost OPS (Plantower PMS7003), and deployed in each of the 15 municipalities (in three schools in 11, in two schools in 3, and in four schools in 1 of the municipalities). We analysed the data set acquired in the period from April 2022 to June 2023. To ensure uninterrupted reporting, reliability was increased by adding redundant nodes, i.e., three nodes with PM sensors placed at each of the measuring sites (location) measured particulate matter pollution in the outdoor school environment (Figure 1b). 

The protocol for the outdoor placement of network nodes at schools had the following requirements: not to face a traffic-dense street or a parking lot that would interfere with the measurements in irregular intervals; not to be in the immediate vicinity of trees that would locally reduce air pollution; and not to be at the height above the first-floor level.

The performance metrics of the sensor nodes were checked by periodic calibration, the details of which can be found in Appendix A. After each calibration, coefficients and measurement uncertainties were obtained and quantified by Root Mean Square Error (RMSE) [5], which was an average of 6.50 µg/m^3^ and 12.95 µg/m^3^ after initial calibration for PM_2.5_ and PM_10_, respectively. Parameters of linear calibration for every sensor were calculated from measured values for the period when low-cost sensors were collocated near the SEPA automatic monitoring station equipped with a Grimm EDM 180 equivalence PM monitor. Average hourly values were obtained according to minute values from all available sensors from one school. It was necessary to have a data coverage of 75% for an hourly datapoint to be valid. Similarly, a data coverage of 75% was needed to obtain a valid daily average.

### 2.2. Comparison with Data from the State AQ Monitoring Network

**Figure 2:**
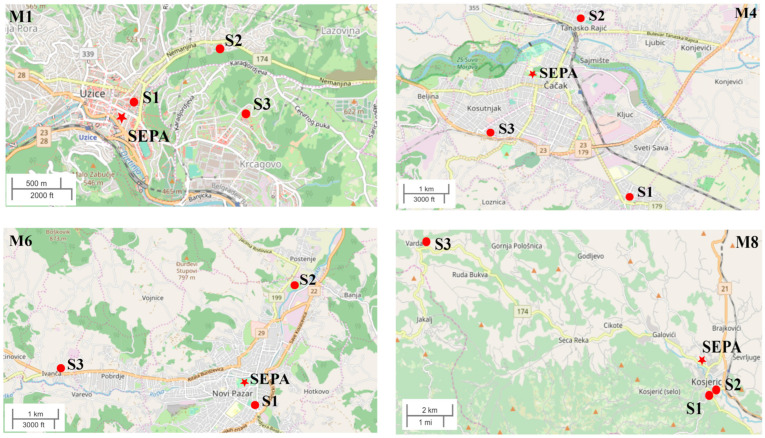
Maps of low-cost sensor locations and SEPA for municipalities M1, M4, M6, and M8. Points present schools and stars present SEPA locations.

In this study, hourly PM_10_ and PM_2.5_ data were obtained via the State Network run by the Serbian Environmental Protection Agency (SEPA), http://www.amskv.sepa.gov.rs/ (accessed on 19 August 2023). Automatic monitoring stations belonging to the State AQ Monitoring Network equipped with Grimm EDM 180 monitor exist in 4 out of the 15 municipalities in which low-cost sensors are located as follows: M1, M4, M6, and M8. The maps of SEPA and low-cost sensor sites are given in Figure 2, and Table 1 presents the distance of each school from the SEPA location in each of these four cities. The simultaneous data obtained from SEPA and the low-cost sensors are available for the period of May 2022–June 2023.

## 3. Results and Discussion

### 3.1. PM_2.5_ and PM_10_ Measurements by SEPA Stations and Low-Cost Sensor Networks

**Figure 3:**
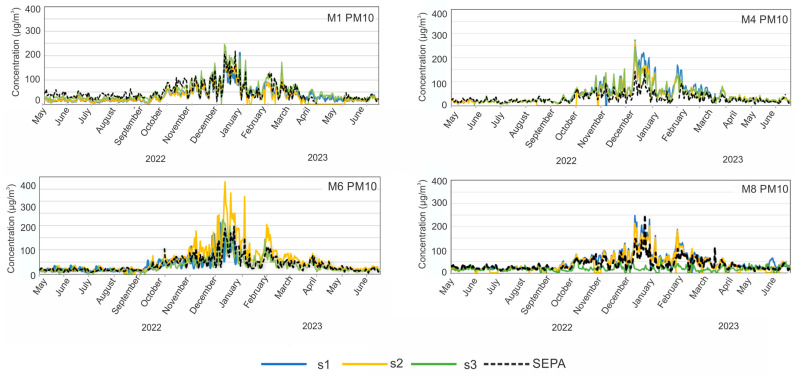
Time series of hourly PM_10_ data reported for each of the community monitoring locations over the study period. SEPA data are presented as a dashed black colour, and data from schools s1, s2, and s3 are presented as blue, yellow, and green, respectively.

**Figure 4:**
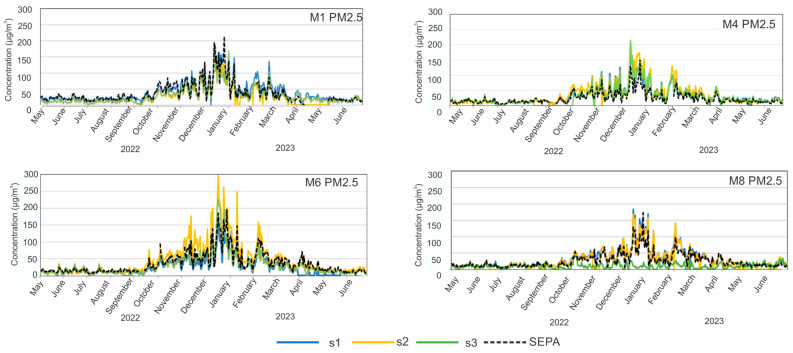
Time series of hourly PM_2.5_ data reported for each of the low-cost monitoring locations over the study period. SEPA data are presented as a dashed black colour, and data from schools s1, s2, and s3 are presented as blue, yellow, and green, respectively.

During the campaign, data derived from the low-cost PM sensors were compared with the results obtained from the SEPA air quality monitoring station. Time series plots for the SEPA station and each low-cost sensor location within each municipality are provided in Figure 3 and Figure 4. They show how low-cost time series (lines in colours) vary in relation to the data from the SEPA instrument (dashed black line). In general, the concentrations of PM_10_ as well as PM_2.5_ are elevated during the winter months. As can be seen in Figure 3, the exceedances of PM_10_ over 50 µg/m^3^ occur during the period October 2022–January 2023, but for M1, they occur during the whole period. The maximum concentration of PM_10_ was in M6, reaching over 350 µg/m^3^. The time series for PM_2.5_ also show large values for M1 and M4, with maximums of more than 200 µg/m^3^.

For each of the four municipalities with SEPA stations, we can compare the PM_10_ and PM_2.5_ concentrations to those obtained with low-cost sensor networks at three locations in every city. For M1, the SEPA results are in agreement with those obtained by the low-cost sensors at all three locations (locations can be seen in Figure 1), even if some locations are around 1 km from SEPA. On the other hand, for municipalities M4 and M6, during the heating season, SEPA showed much lower PM_10_ concentrations than the low-cost sensors (max difference of around 200 µg/m^3^). For M4, the SEPA station is in the urban area of the city and the other three locations are more than 1.5 km away from the SEPA station (Figure 1). For M6, the measured levels have similar trends; however, at one of the sites (s2), the concentration reported by the LCS network is much higher than SEPA and the other sites in M6, potentially indicating a strong local influence of individual heating. At M8, two locations of low-cost sensors are in agreement with the SEPA results, but one location displayed totally different PM_10_ results because it is far away from the urban area. Similar results can be seen for PM_2.5_ in Figure 4, while for M1 low-cost sensors, the data are in the best agreement with SEPA. In addition, it can be observed that in all four municipalities, a better agreement between low-cost sensors and SEPA occurs at lower concentrations, indicating better sensor performance for lower PM concentrations. 

### 3.2. PM_10_ and PM_2.5_ Measurements by Low-Cost Sensor Networks

**Figure 5:**
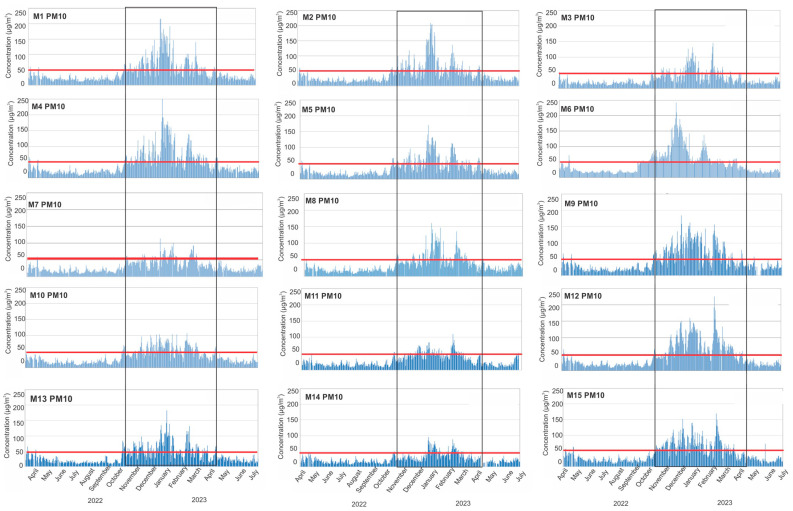
Time series of hourly PM_10_ data averaged per three (most commonly) measurement sites within the municipality over the study period. Grey rectangles represent the heating season, and the red line is the limit for PM_10_ of 50 µg/m^3^.

**Figure 6:**
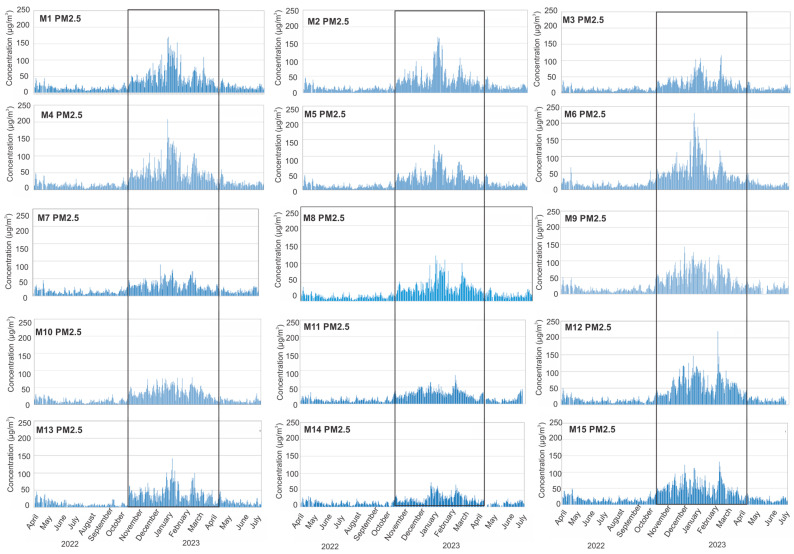
Time series of hourly PM_2.5_ data averaged per three (mostly) measurement sites within the municipality over the study period. Grey rectangles represent the heating season.

The averaged time series for PM_10_ and PM_2.5_ across the whole measured period in every municipality within the low-cost sensor network are displayed in Figure 5 and Figure 6. They indicate that the concentrations of PM_10_ and PM_2.5_ are much higher during the heating season—October 2022 to April 2023. 

The exceedances of PM_10_ concentrations over 50 µg/m^3^ occur during the heating period October 2022–April 2023 in almost all municipalities (see Figure 5). The maximum concentrations can be seen in municipalities M1, M2, and M4, where they were more than 200 µg/m^3^ during the winter months. On the other hand, during the summer months, there were almost no days of exceedance. 

When we observe the time series for PM_2.5_, the maximum concentrations occur in M1, M4, M6, and M12 during the winter months (Figure 6) when the mass concentrations were over 200 µg/m^3^ for one or two days. The smallest values of PM_2.5_ are in M7, M10, M11, and M14, which is probably associated with locations where sensors collected PMs in rural areas, similar to PM_10_ concentrations in these municipalities.

For an indication of the current status of urban air pollution, the European Air Quality Index (AQI) [39] was used. We calculated the percentage of days during the examined period according to each category as follows:

- PM_10_: very good 0–20 µg/m^3^; good 20–40 µg/m^3^; medium 40–50 µg/m^3^; poor 50–100 µg/m^3^; very poor 100–150 µg/m^3^; and extremely poor 150–1200 µg/m^3^.
- PM_2.5_: very good 0–10 µg/m^3^; good 10–20 µg/m^3^; medium 20–25 µg/m^3^; poor 25–50 µg/m^3^; very poor 50–75 µg/m^3^; and extremely poor 75–800 µg/m^3^.

**Figure 7:**
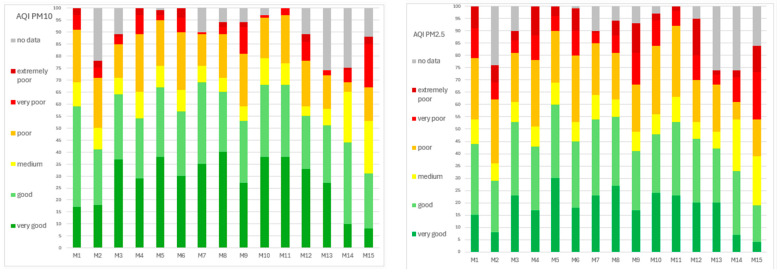
Percentage of days with each of the 6 AQI categories for PM_10_ and PM_2.5_ in all 15 municipalities.

Figure 7 shows the percentage of days during the whole period (April 2022–June 2023) of every AQI category in each city. 

Poor-extremely poor AQI for PM_10_ was detected in the range of 1 to 21% of days. The highest value of poor-extremely poor air was in municipality M15, and only 1% of such highly polluted days occurred in M7. Poor-extremely poor AQI for PM_2.5_ was detected in the range of 5 to 30% of days, while in 3/15 municipalities, i.e., M9, M12, and M1, it was in the range of 25–30% of days. 

However, very good-good PM_10_ values were all in the range of 31–69% of days, with more than 60% of days in 6/15 municipalities (M3, M5, M7, M8, M10, and M11), while 31% of days, which was the lowest number of days, was identified in M15. Regarding AQI for PM_2.5_, for very good–good, it varied through municipalities in the range from 19% to 60% of days, the lowest observed in M15 and the highest in M5. In 5/15 municipalities, more than 50% of days were in the very good–good AQI PM_2.5_.

**Figure 8:**
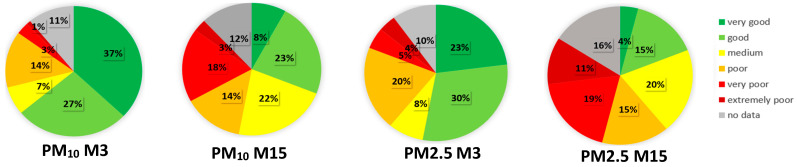
Pie charts with the percentage of days for each of the 6 AQI categories for PM_10_ and PM_2.5_ in M3 and M15.

In Figure 8, pie charts are given for one of the most polluted cities, Prijepolje (M15), located at the River Lim surrounded by mountains, and one of the less polluted cities, Kruševac (M3), which is in the valley of Zapadna Morava River, according to the analysis of AQI categories of PMs for 15 months (6 months of heating and 9 months of non-heating).

With this study, we confirmed that having monitoring sites equipped even with low-cost sensor devices in one municipality can help to identify PM daily concentrations, pollution episodes, and even diurnal variations, especially in cities in which there is no PM monitoring in the framework of a state or local monitoring network. For instance, municipalities M12, M11, and M10 have a large percentage of days with poor AQI categories for PM_10_ and PM_2.5_, and in these municipalities, there are no automatic monitoring stations.

An important aspect of this kind of study is the ability of low-cost sensors to provide the public with more immediate and relevant information via websites and services that can warn individuals when air pollution levels are above thresholds. The outcome of this study can raise the awareness of citizens and local authorities about PM pollution and the necessity for performing more studies, online monitoring, and modelling in the 15 municipalities that participated in this study.

## 4. Conclusions

In this study, a large network of low-cost particulate matter monitors was deployed at 43 locations in 15 municipalities in central and western Serbia, which measured PM_10_ and PM_2.5_ for a period of 15 months, from April 2022 to June 2023. In almost all the municipalities, the main source of pollution is individual heating and associated facilities, while in a few municipalities, there are well-known industrial activities. Traffic intensity also contributes in some sites where the diffusion of pollution is hindered by local orography.

Based on the time series of PM concentration in municipalities 1 and 4, which have both SEPA and low-cost sensor PM monitors, we concluded that the levels for PM_2.5_ and PM_10_ are comparable to the SEPA results and are generally very similar across schools in the same municipality, with some exceptions, which are due to the presence of strong local sources or differences in population densities between urban and rural areas. In all 15 municipalities, the maximum concentrations of PM_2.5_ and PM_10_ were observed in the winter season, while during the summer months, the PM concentrations were significantly lower. In almost all municipalities, the most polluted months were December and/or January. PM_10_ 24 h concentration in each municipality exceeded limit values of 50 µg/m^3^. 

Looking at the distributions of AQI categories of PMs for the whole period, the highest percentage of poor–extremely poor AQI were in M7 and M12 for PM_10_ and in M1, M4, M6, and M7 for PM_2.5_. On the other hand, very good to medium AQI PM_10_ values were the highest in M10 and M11, while for PM_2.5_, they were best in M5 and M11.

The significant result of this study is the ability of low-cost sensors to provide the public with more information about air pollution levels, especially in municipalities where offline and online monitoring of PM_10_ and PM_2.5_ within local- and state-level networks does not exist. Furthermore, in the UNICEF initiative, the importance of raising awareness about ambient air pollution is one of the main goals. Furthermore, since sensors were placed in the outdoor micro-environment, this study has hopefully helped in raising the awareness of school children about this topic.

## Figures and Tables

**Table 1:** Distances of low-cost sensor locations from SEPA stations in four cities.

Municipality	School	Distance from SEPA
M1	S1	0.175 km
	S2	1.00 km
	S3	1.05 km
M4	S1	1.54 km
	S2	1.50 km
	S3	3.31 km
M6	S1	0.87 km
	S2	2.47 km
	S3	4.75 km
M8	S1	0.87 km
	S2	1.08 km
	S3	12.90 km

**Figure A1:**
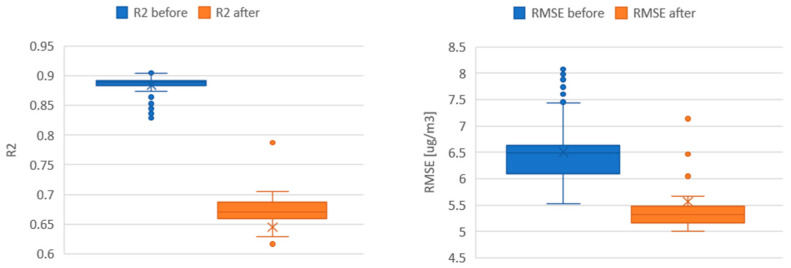
Summary statistics of the PM_2.5_ calibration performance of the LCS nodes before deployment (denoted as R^2^ before and RMSE before) and after (re)calibration (denoted as R^2^ after and RMSE after).

## Data Availability

Data are contained within the article.

## Appendix A

Before deployment in the 15 municipalities, all sensor network nodes were subject to an extensive collocation campaign that was conducted at the SEPA station in Belgrade and lasted for 3 weeks (4–28 February 2022). Summary statistics of the meteorological and air pollution conditions during the collocation campaign are given in Table A1.

**Table A1:** Summary statistics of the meteorological and air pollution conditions during the first collocation campaign, SEPA station Belgrade, 4–28 February 2022.

	PM_1_ [μg/m^3^]	PM_2.5_ [μg/m^3^]	PM_10_ [μg/m^3^]	SO_2_ [μg/m^3^]	NO_2_ [μg/m^3^]	T [°C]	RH [%]
2nd perc.	2.79	3.50	4.63	3.16	7.95	2.29	33.23
98th perc.	86.30	90.36	118.24	27.14	123.21	17.20	89.63
mean	22.03	24.68	35.02	9.98	50.88	8.23	62.04
median	16.28	19.08	27.34	7.55	48.63	7.75	61.14

The calibration coefficients for all sensor nodes were derived based on this initial collocation campaign before deployment [40]. After that, the sensors were deployed in the 15 municipalities where they produced a calibrated data stream. Box plots of performance metric summary statistics (R^2^ and RMSE) are given in Figure A1 (denoted as before deployment). Since we used univariate linear regression, the impact of the linear regression term is on the scale of the predictions. Furthermore, the intercept parameter of the linear regression model unifies the response across the sensor fleet in conditions of low pollution.

Recalibration efforts are a needed part of a metrologically sound approach to low-cost sensor network deployment. These recalibration efforts have several purposes. Firstly, since the long-term deployment will span several seasons, calibration coefficients derived during one season could be inadequate for another season. That is why recalibration should be performed once the meteorological conditions have changed significantly. Secondly, sensor performance may degrade over time. It is possible to quantify such drops in performance by performing a new calibration.

The new (re)calibration campaign was conducted in the period from 11 April to 11 May 2023. Summary statistics of the meteorological and air pollution conditions during the (re)calibration collocation campaign are given in Table A2.

**Table A2:** Summary statistics of the meteorological and air pollution conditions during the second collocation campaign, SEPA station Čačak, 11 April–11 May 2023.

	PM_1_ [μg/m^3^]	PM_2.5_ [μg/m^3^]	PM_10_ [μg/m^3^]	SO_2_ [μg/m^3^]	NO_2_ [μg/m^3^]	T [°C]	RH [%]
2nd perc.	/	3.27	4.40	5.60	3.38	4.00	30.55
98th perc.	/	44.16	59.93	21.99	38.92	22.61	92.24
mean	/	15.32	21.15	8.22	11.70	13.28	64.66
median	/	13.50	18.80	6.85	9.46	13.16	68.79

Note that the currently accepted best practice is to perform collocation next to the reference station, thus ensuring the traceability of the collocated instrument. However, another requirement was also imposed on the sensor network to ensure uninterrupted continuous reporting. To meet both requirements, the method that was used for calibration was a combination of a collocation method (as used in the initial calibration when it was possible to collocate all available instruments) and a transfer of calibration (in order to transfer the new calibration coefficients to the instrument that were not collocated but instead stayed at the deployment location in order to ensure uninterrupted reporting).

Furthermore, since calibrated devices can be used to transfer calibration, transfer coefficients are also needed. These were calculated based on the most recent month (March 2023) when the devices were collocated at their deployment locations. In summary, for each collocated device, 1-hop calibration and 1-hop transfer coefficients were calculated, as well as 2-hop transfer/calibration coefficients. Here, the term “hop” is used to name a process of a single univariate linear transformation on the data, for which the transformation coefficients were derived using collocation (either between the low-cost sensor and the reference or between the two low-cost sensors). This procedure results in updated coefficients for all LCS nodes in each school, where a collocated unit will have new coefficients obtained via 1-hop linear calibration, and the remaining deployed units will have new coefficients obtained via 2-hop linear calibration, which are obtained by composition of transfer and calibration.

As illustrated in Figure A1, with the approach to calibration and recalibration used, which is also recommended for low-cost sensor networks by WMO [41], it is only possible to observe how the performance changed over the period of the deployment. However, it is not possible to have a more detailed picture of fine/gradual temporal changes in uncertainty.
